# Fruit and Vegetable Intake and Body Mass Index in a Large Sample of Middle-Aged Australian Men and Women

**DOI:** 10.3390/nu6062305

**Published:** 2014-06-17

**Authors:** Karen Charlton, Paul Kowal, Melinda M. Soriano, Sharon Williams, Emily Banks, Kha Vo, Julie Byles

**Affiliations:** 1 School of Medicine, Faculty of Science, Medicine and Health, University of Wollongong, New South Wales 2522, Australia; 2 Research Centre for Gender, Ageing and Health, University of Newcastle, New South Wales 2308, Australia; E-Mails: kowalp@who.int (P.K.); kha.vo@newcastle.edu.au (K.V.); julie.byles@newcastle.edu.au (J.B.); 3 WHO Multi-Country Studies, World Health Organization, Geneva CH-1211, Switzerland; 4 Department of Pharmacy Practice, University of Illinois at Chicago, Chicago, IL 60612, USA; E-Mail: melinda_soriano@yahoo.com; 5 Department of Anthropology and Center on Aging and the Life Course, Purdue University, West Lafayette, IN 47907, USA; E-Mail: srw@purdue.edu; 6 College of Medicine, Biology and Environment, Australia National University, Canberra ACT 0200, Australia; E-Mail: Emily.Banks@anu.edu.au

**Keywords:** fruit, vegetables, BMI, dietary guidelines, obesity

## Abstract

Dietary guidelines around the world recommend increased intakes of fruits and non-starchy vegetables for the prevention of chronic diseases and possibly obesity. This study aimed to describe the association between body mass index (BMI) and habitual fruit and vegetable consumption in a large sample of 246,995 Australian adults aged 45 + year who had been recruited for the “45 and Up” cohort study. Fruit and vegetable intake was assessed using validated short questions, while weight and height were self-reported. Multinomial logistic regression was used, by sex, to assess the association between fruit and vegetable intake and BMI. Compared to the referent normal weight category (BMI 18.5 to 24.9), the odds ratio (OR) of being in the highest vegetable intake quartile was 1.09 (95% confidence interval (CI) 1.04–1.14) for overweight women (BMI 25.0–29.9) and 1.18 (95% CI 1.12–1.24) for obese women. The association was in the opposite direction for fruit for overweight (OR 0.85; 95% CI 0.80–0.90) and obese women (OR 0.75; 95% CI 0.69–0.80). Obese and overweight women had higher odds of being in the highest intake quartile for combined fruit and vegetable intake, and were more likely to meet the “2 and 5” target or to have five or more serves of fruit and vegetables per day. In contrast, overweight men were less likely to be in high intake quartiles and less likely to meet recommended target of 5 per day, but there was no consistent relationship between obesity and fruit and vegetable intake. Underweight women and underweight men were less likely to be in the highest intake quartiles or to meet the recommended targets. These data suggest that improving adherence to dietary targets for fruit and vegetables may be a dietary strategy to overcome overweight among men, but that overweight and obese women are already adhering to these targets. The association between fruit and vegetable intake and underweight in adults suggests that improving fruit and vegetables intakes are important for the overall dietary patterns of people in this group.

## 1. Introduction

In 2005, the World Health Organization estimated 1.6 billion adults were overweight and 400 million obese [1]. Worldwide, obesity estimates are projected to increase to 2.3 billion adults being overweight and 700 million obese by 2015, further supporting the need to identify public health strategies for sustainable weight management.

International agencies have identified the level of evidence to be convincing for a reduced risk between fruit and non-starchy vegetable intake and cardiovascular disease [2] and probable for a reduced risk for some cancers, diabetes and obesity, as well as for the prevention of several micronutrient deficiencies [2,3]. However, the contribution of inadequate fruit and vegetable intake to development of overweight and obesity is difficult to quantify since reliable dietary patterns are not readily available [4].

In 2011, the Australian population ranked fifth highest in the prevalence of obesity among OECD countries, which will translate into substantially higher health and non-health care costs attributed to obesity than previously estimated [5,6,7]. Unlike in some countries such as Canada, England, Italy, Korea, Spain and the United States where obesity and overweight have been virtually stable, or have grown modestly, over the past five years, rates have increased by a further 2%–3% in Australia. A global shift to more energy-dense foods and physical inactivity is linked to an increase in the prevalence of obesity [8]. The 2010 Global Burden of Disease Study estimates 16,140 deaths per year attributed to low fruit and vegetable intakes in Australia and considerable morbidity attributable to low fruit and low vegetable intakes [9]. Australia’s government funded Go For 2 & 5^®^ fruit and vegetable social marketing campaign (“Go for 2 & 5”) [10,11] has been demonstrated to be successful in increasing population awareness of this recommended number of servings of fruit and vegetables and resulted in a significant increase in vegetable (but not fruit) consumption over a three year period [12].

Women generally consume more fruit and vegetables than men across many countries and cultures, yet almost universally have higher levels of morbidity at the population level but have lower cardiovascular risk and premature mortality [13,14,15,16]. Gender may also potentially moderate the relationship between fruit and vegetable consumption and body weight [17]. The relationship between fruit and vegetable intake and various health outcomes should therefore be assessed for men and women separately. Additionally, much of the existing literature investigating the relationship between fruit and vegetable consumption and body weight have analyzed both as a combined variable (*i.e*., meeting at least 5 servings of fruit and vegetables), rather than examining the effect of each separately [17].

The objective of this study was to investigate the association between fruit and vegetable intake and body weight in a large sample of Australian men and women aged 45 years and over in New South Wales, Australia. A secondary objective was to estimate the proportion of Australians meeting the recommended daily intakes of fruit and vegetables [4].

## 2. Experimental Section

### 2.1. Study Population

The Sax Institute’s 45 and Up Study is a population based cohort study in the state of New South Wales (NSW), Australia. Prospective participants were randomly sampled from the enrolment database of Medicare Australia, a universal health insurance scheme which provides near complete coverage of the population. Permanent residents of Australia, except Norfolk Island residents, are eligible for a Medicare card that entitles them to free in-hospital treatment (public hospitals), subsidized treatment for services out-of-hospital, as well as access to a subsidized Pharmaceutical Benefits Scheme for medicines. People 80+ years of age and residents of rural and remote areas were oversampled. A total of 267,153 participants joined the study by completing a baseline questionnaire (between January 2006 and December 2009) and provided signed consent for follow-up and linkage of their information to routine health databases. About 18% of those invited participated and participants included about 10% of the NSW population aged 45 years and over. The study, as described in detail elsewhere, provides information regarding exposures and outcomes that influence public health interventions in an ageing population [18]. The present analysis included data from the full baseline sample. The conduct of the 45 and Up Study was approved by the University of New South Wales Human Research Ethics Committee (HREC). Use of the data for the present analysis was approved by The Sax Institute (Project number 09023, approved 21 December 2009).

### 2.2. Materials

Variables for the study were obtained from the self-reported baseline questionnaire. Vegetable consumption patterns were obtained from the question, “About how many serves of vegetables do you usually eat each day?” For reference, a “serve” as written within the survey instruments is equivalent to a traditional “serving”. Vegetable intake was reported as the number of servings of cooked and raw vegetables consumed each day, or else coded as “I don’t eat vegetables”. A serving of vegetables was defined as half a cup of cooked vegetables, including potatoes, or one cup of salad. Habitual fruit intake was assessed from the question, “About how many serves of fruit or glasses of fruit juice do you usually have each day?” Fruit intake was reported as the number of servings of fruit each day, or coded as “I don’t eat fruit.” A serving of fruit was defined as one medium piece or two small pieces of fresh fruit, or one cup of diced fruit. Fruit juices were not considered to be fruit for the purpose of this analysis. Fruit intakes greater than four servings per day and vegetable intakes larger than seven servings per day were set to missing as this was considered to be excessive with a high potential for misreporting (including 1.1% missing either). Fruit intake of less than two servings daily or vegetable intake of less than five servings daily were classified as “low” or insufficient, according to the recommendations of the Australian Department of Health and Ageing [10]. Intake quartiles were generated based on total counts for men and women separately, with respect to daily fruit and vegetable servings. A binary indicator designated participants who satisfied the overall recommendation of two or more servings of fruit and five or more servings of vegetables per day. A binary indicator specified participants who satisfied the WHO recommended intake of “5 serves per day” of combined fruit and vegetables [2,11]. Body mass index (BMI) was calculated as weight in kilograms divided by the square of the height in meters, with both variables being self-reported in the questionnaire. The underweight category was defined as BMI < 18.5, normal weight as BMI 18.5 to 24.9, overweight as BMI 25.0 to 29.9 and obese as BMI ≥ 30 [8]. Sociodemographic variables, such as age, sex, income level, highest education level, marital status, work hours per week, location of residence, country of birth were included as independent variables, along with confounders such as physical activity levels, alcohol consumption and smoking.

### 2.3. Statistical Analysis

Logistic regression was used to assess the relationship between fruit and vegetable intake and BMI for men and women separately, with a multinomial logistic approach used for the quartile outcomes. Analyses were adjusted for the sociodemographic variables and confounders listed above and results were reported as adjusted odds ratios (ORs) with 95% confidence intervals (CIs). Analyses were conducted using STATA 11.0 (Statacorp LP, College Station, TX, USA), and SAS V9.2 (SAS Institute Inc., Cary, NC, USA).

## 3. Results

Sociodemographic characteristics of the 246,995 participants with complete data for BMI are presented in Table 1. The mean age (and standard deviation) was 63.8 years (11.1) for men and 61.8 years (11.1) for women. Men were more likely to have achieved a post-school qualification (63.8% *versus* 48.9% respectively) and were more likely to be partnered (80.1% *versus* 70.1%).

Table 2 presents the fruit and vegetable intake descriptors and BMI categories. Over half of study participants reported eating two or more servings of fruit per day and nearly one quarter reported eating five or more servings of vegetables daily, with 15.1% of participants satisfying the “Go for 2 & 5” guideline (Table 2). Sex differences exist in fruit consumption, vegetable consumption, the combination of fruit and vegetable consumption, and body mass index (Table 2). On average, men ate less fruit (mean 1.6 servings/day) and less vegetables (mean 2.9 servings/day) than women (mean 1.9 servings of fruit and 3.7 servings of vegetables per day) (*p* < 0.0001 for both comparisons). Women were nearly twice as likely to satisfy or exceed the “2 fruit and 5 vegetable” recommendation (19.9% of women *versus* 9.7% of men). Men were more likely to be overweight or obese compared to women (69.0% *versus* 57.1% respectively).

**Table 1:** Socio-demographic characteristics of participants, by sex.

	Men	Women	Total
	(*n* = 116,029)	(*n* = 130,966)	(*n* = 246,995)
	(%)	(%)	(%)
**Age (year)**			
45–49	(9.8)	(13.1)	(11.6)
50–59	(30.7)	(35.9)	(33.5)
60–69	(29.3)	(27.5)	(28.4)
70–79	(18.5)	(14.2)	(16.2)
80–89	(10.9)	(8.5)	(9.6)
90+	(0.7)	(0.8)	(0.8)
**Place of Residence**			
Major city	(46.3)	(44.2)	(45.2)
Inner regional	(34.4)	(35.9)	(35.2)
Outer regional	(17.4)	(17.9)	(17.7)
Remote	(1.9)	(2.0)	(2.0)
**Highest Educational Qualification**			
No qualification	(10.5)	(12.0)	(11.3)
School leaving certificate	(24.8)	(37.8)	(31.7)
Trade/certificate	(37.8)	(26.8)	(32.0)
University	(25.3)	(22.1)	(23.6)
Missing	(1.5)	(1.4)	(1.4)
**Marital Status**			
Single	(5.9)	(5.4)	(5.7)
Married	(74.7)	(64.7)	(69.4)
Partner	(5.5)	(5.4)	(5.4)
Widowed	(4.8)	(11.8)	(8.5)
Divorced	(5.5)	(9.5)	(7.6)
Separated	(2.7)	(2.9)	(2.8)
Missing	(1.0)	(0.4)	(0.6)

**Table 2:** Fruit and vegetable intake and Body Mass Index (BMI), by sex.

	Men *n* = 116,029 *n* (%)	Women *n* = 130,966 *n* (%)	Total *n* = 246,995 *n* (%)
**Fruit Intake**			
<2 servings/daily (Low)	54,546 (47.0)	44,397 (33.9)	98,943 (40.1)
≥2 servings/daily (Adequate)	51,452 (44.3)	77,876 (59.5)	129,328 (52.4)
Missing *	10,031 (8.6)	8693 (6.6)	18,724 (7.6)
**Vegetable Intake**			
<5 servings/daily (Low)	86,664 (74.7)	77,878 (59.5)	164,542 (66.6)
≥5 servings/daily (Adequate)	19,623 (16.9)	37,442 (28.6)	57,065 (23.1)
Missing *	9742 (8.4)	15,646 (11.9)	25,388 (10.3)
**Fruit and Vegetable Intake (“2** **&** **5”)**			
Low (<2F and/or <5V)	88,171 (76.0)	84,331 (64.4)	172,502 (69.8)
Adequate (at least 2F and 5V)	11,308 (9.7)	26,101 (19.9)	37 409 (15.1)
Missing *	16,550 (14.3)	20,534 (15.7)	37,084 (15.0)
**Body Mass Index**			
Underweight (BMI <18.5)	867 (0.7)	2494 (1.9)	3361 (1.4)
Normal weight (BMI 18.5–24.9)	35,118 (30.3)	53,781 (41.1)	88,899 (36.0)
Overweight (BMI 25–29.9)	54,719 (47.2)	44,346 (33.9)	99,065 (40.1)
Obese (BMI ≥ 30)	25,325 (21.8)	30,345 (23.2)	55,670 (22.5)

* Includes >4 serves of fruit a day or >7 serves of vegetable a day.

**Figure 1:**
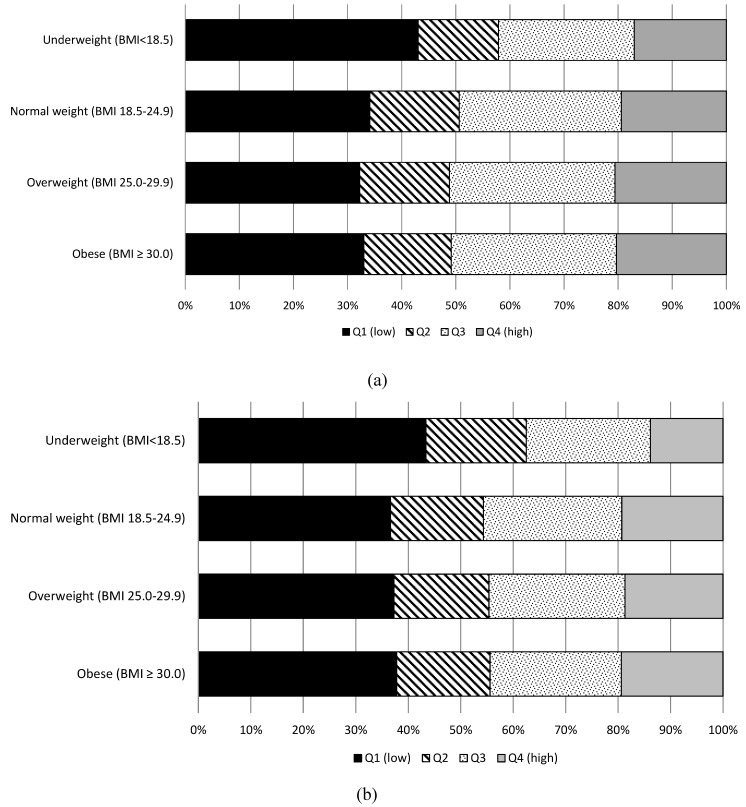
(**a**) Distribution of fruit and vegetable intake for women determined by overall intake quartiles, according to BMI; (**b**) Distribution of fruit and vegetable intake for men determined by overall intake quartiles, according to BMI.

Figure 1a,b present the distribution of combined fruit and vegetable intake quartiles for women and men separately. Patterns by intake quartiles were not considerably different by BMI category for either sex, with the exception of underweight people, who were more likely to be in the lower intake quartile.

**Figure 2:**
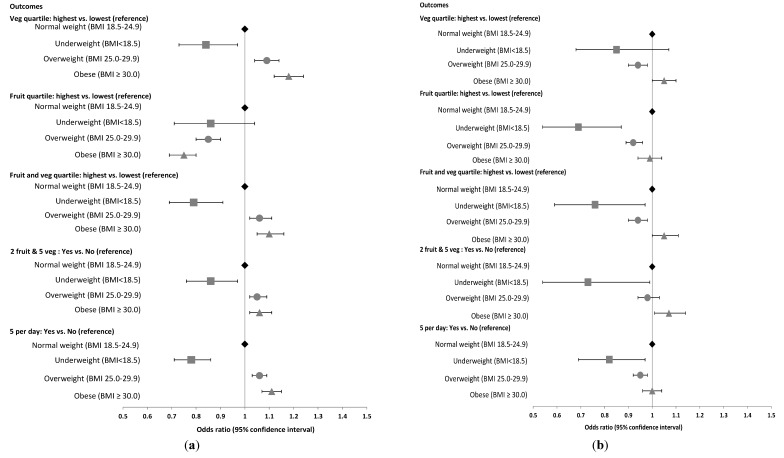
(**a**) Women: Adjusted * Odds Ratios and 95% Confidence Intervals for fruit and vegetable intake groupings, by BMI group; (**b**) Men: Adjusted * Odds Ratios and 95% Confidence Intervals for fruit and vegetable intake groupings, by BMI group. * Adjusted for age, education level, marital status, location of residence, income level, country of birth, physical activity, smoking, and alcohol intake.

Figure 2a,b graphically present the adjusted odds ratios (ORs) for fruit and vegetable consumption outcomes with respect to BMI category, for women and men separately. The first three results relate to the highest *versus* lowest quartile comparisons for vegetable intake fruit intake and combined fruit and vegetable intakes (lowest quartile was the reference group). The last two results include the “2 Fruit & 5 Vegetable” outcome and the “5 per day” combined fruit and vegetable outcome.

Compared to the referent normal weight category (BMI 18.5 to 24.9), women who were underweight were less likely to be in the highest vegetable consumption quartile (OR 0.84; 95% CI 0.73–0.97) while women who were overweight or obese were significantly more likely to be in the highest vegetable consumption quartile (OR 1.09; 95% CI 1.04–1.14) and OR 1.18; 95% CI 1.12–1.24), respectively) (Table 3). The association was in the opposite direction for fruit, with obese women being 25% less likely to be in the highest intake quartile (OR 0.75; 95% CI 0.69–0.80), and overweight women 15% less likely (OR 0.85; 95% CI 0.80–0.90). Obese women had higher odds of being in the highest intake quartile for combined fruit and vegetable intake (OR 1.10; 95% CI 1.05–1.16), and were more likely to meet either “2 & 5” (OR 1.06; 95% CI 1.02–1.11) or “5 per day” (OR 1.11; 95% CI 1.07–1.15). Underweight women were less likely than normal weight women to meet both the “2 & 5” (OR 0.86; 95% CI 0.76–0.97) or “5 per day” targets (OR 0.78; 95% CI 0.71–0.86), and were less likely to be in the highest quartile for vegetable intake (OR 0.84; 95% CI 0.73–0.97) or fruit and vegetable combined intake (OR 0.79; 95% CI 0.69–0.91).

**Table 3:** Odds ratio of being in highest fruit and vegetable quartiles according to Body Mass Index (BMI) category, by sex.

	Highest Vegetable Quartile	Highest Fruit Quartile	Highest F & V Quartile	2F & 5V = Yes	5 Per Day = Yes
	OR (95% CI)	OR (95% CI)	OR (95% CI)	OR (95% CI)	OR (95% CI)
**BMI categories—Women**					
Normal weight * (BMI 18.5–24.9)	1	1	1	1	1
Underweight (BMI < 18.5)	0.84 (0.73; 0.97)	0.86 (0.71; 1.04)	0.79 (0.69; 0.91)	0.86 (0.76; 0.97)	0.78 (0.71; 0.86)
Overweight (BMI 25.0–29.9)	1.09 (1.04; 1.14)	0.85 (0.80; 0.90)	1.06 (1.02; 1.11)	1.05 (1.02; 1.09)	1.06 (1.03; 1.09)
Obese (BMI ≥ 30.0)	1.18 (1.12; 1.24)	0.75 (0.69; 0.80)	1.10 (1.05; 1.16)	1.06 (1.02, 1.11)	1.11 (1.07; 1.15)
**BMI categories—Men**					
Normal weight * (BMI 18.5–24.9)	1	1	1	1	1
Underweight (BMI < 18.5)	0.85 (0.68; 1.07)	0.69 (0.54; 0.87)	0.76 (0.59; 0.97)	0.73 (0.54; 0.99)	0.82 (0.69; 0.97)
Overweight (BMI 25.0–29.9)	0.94 (0.90; 0.98)	0.92 (0.89; 0.96)	0.94 (0.90; 0.98)	0.98 (0.94; 1.03)	0.95 (0.92; 0.98)
Obese (BMI ≥ 30.0)	1.05 (1.00; 1.10)	0.99 (0.94; 1.04)	1.05 (1.00; 1.11)	1.07 (1.01; 1.14)	1.00 (0.96; 1.04)

* Reference category.

The results are different for men. Compared to the referent normal weight group, overweight men were less likely to be in the highest vegetable (OR 0.94; 95% CI 0.90–0.98), fruit (OR 0.92; 95% CI 0.89–0.96) and combined fruit and vegetable intake quartiles (OR 0.94; 95% CI 0.90–0.98) and less likely to have five servings of fruit and vegetables per day (OR 0.95; 95% CI 0.92–0.98) (Table 3). Obese men were more likely to meet the “2 & 5” target (OR 1.07; 95% CI 1.01–1.14) than normal weight men. Underweight men were less likely than normal weight men to meet either the “2 & 5” (OR 0.73; 95% CI 0.54–0.99) and “5 per day” targets (OR 0.82; 95% CI 0.69–0.97) and less likely to be in the highest quartile for fruit intake (OR 0.69; 95% CI 0.54–0.87) and combined fruit and vegetable intake (OR 0.76; 95% CI 0.59–0.97).

## 4. Discussion

Fruits and vegetables, due to their high water and fiber content, and relatively low energy content have an important role in weight management due to their effect on satiety and reduced hunger [19]. Increased fruit, vegetables and dietary fiber intake has been reported to be independently associated with greater weight loss among overweight middle-aged adults [20]. However, the weight loss effects associated with dietary components in fruit and vegetables appear to be related to the food matrix, rather than the bulking properties of fiber content of these foods. [21] This cross-sectional analysis of 246,995 Australian adults has demonstrated that overweight and obese women were more likely than their normal weight counterparts to consume high intakes of vegetables, combined fruits and vegetables, are more likely to meet daily intake recommendations. However, overweight men were less likely than normal weight men to meet recommendations for fruit and vegetable targets.

Our findings in women are not consistent with data from epidemiological studies that have generally reported that increasing vegetable intake is associated with decreased weight gain [22], although different age groups have been studied [20,23,24] and results are not consistent for children and adolescents [25]. Our results are also not in line with previous data that analyzed a similarly large dataset of adults from the United States Behavioral Risk Factor Surveillance Survey [26]. In that study, an inverse relationship between body mass index and consumption of fruits and vegetables (using a threshold of 5 servings daily) was found. An explanation for the opposite direction in Australian middle-aged women could be related to excessive consumption of both nutrient dense foods (such as vegetables, and other foods from the five core food groups), as well as foods with high energy density and low nutrient density such as discretionary foods, resulting in energy imbalance and hence weight gain in this population. Lack of physical activity, combined with a diet that includes a high vegetable intake may also be a contributing factor. Further research is needed to determine if this is the case.

Unique to this study was the separate evaluation of fruit and vegetable consumption on body mass index. Opposite directions were seen for fruits, with overweight and obese women being less likely than their normal weight counterparts to have intakes in the highest quartile. This association was also found for overweight, but not obese, men. Evidence related to the association between fruit intake and BMI is inconsistent. The prospective Nurses’ Health Study found increased fruit consumption was associated with a 28% lower risk of weight gain in women with high fruit intake [22,23,24]. Similarly, a Canadian cohort study reported inverse correlations between fruit intake and change in weight, fat and waist circumference [27]; however, two other cohort studies have not reported a protective effect [23,24]. The results indicate unique contributions of fruit intake to managing body weight, and support the need for separation of the effects of fruit and vegetable food groups [17], as shown herein. The positioning of fruit within a particular cuisine and food pattern will influence overall energy density. Men and women may include fruit in their diets in different ways but interpretation of our data is limited by the lack of a comprehensive dietary assessment and information on intake of other food groups. For both men and women, compared to those with a BMI in the normal weight range of 18.5–24.9, those that were underweight were less likely to have higher vegetable intakes, higher fruit intakes and less likely to meet the daily targets for fruit and vegetable intakes which suggests that dietary intervention is needed to address poor dietary patterns in this group.

Only 5.5% of Australian adults had an adequate daily intake of 2 fruit and 5 vegetables servings in this study, suggesting the need to consider strategies to increase fruit and vegetable consumption. It should be noted, this study was undertaken after a social marketing campaign (“Go for 2 & 5”), and would indicate further efforts to encourage fruit and vegetable intake are required. Such efforts might include more marketing of dietary messages, but may also consider additional factors such as availability and price [28,29,30]. Women were more likely to meet both fruit and vegetable guidelines than men (6.5% and 4.5% respectively). Older Australians were more likely to meet the guidelines than younger adults, with 8.8% of persons aged 85 years and over consuming the recommended intake of fruits and vegetables, compared with 3.4% of persons aged 25–34 years. The 2011–2012 nationally representative Australia Health Survey found that 48.5% of Australians aged 18 years and over usually ate two or more servings of fruit per day (meeting the guidelines), while 8.2% usually ate 5 or more servings of vegetables per day (meeting the guidelines) [31]. Our figures suggest higher intakes of both fruits and vegetables in the 45 and Up Study cohort, with 16% of men and 30% of women meeting or exceeding “2 & 5” threshold, consistent with the frequently observed “healthy cohort effect” [30]. However, it should be noted that theoretical and empirical work indicates that the OR estimates here, based on internal comparisons within the cohort, are likely to be reliable and generalisable [30].

The 45 & Up Study collected dietary information that can be used to assess fruit and vegetable intakes against Australian government recommendations of at least two servings of fruit and five servings of vegetables daily. Using the US and UK recommendations of five servings of combined fruits and vegetables intake per day as the threshold, our analysis indicates that 50% of New South Wales adults can be considered to be meeting these recommended daily intakes. Women are more likely to meet recommendations, with 58% of women (compared to 40% of men) consuming adequate amounts. This compares to data from the World Health Survey (2002–2003), in which 77.6% of men and 78.4% of women from mainly low- and middle-income countries consumed less than five servings of fruits and vegetables [32]. Similar questions were employed across the two studies. In the United Kingdom National Diet and Nutrition Survey (2002), 86% of British adults consumed less than the recommended five portions per day [33]. Similarly, in the US, the Behavioral and Risk Factor Surveillance System conducted between 2000 and 2009, identified that 67.5% consumed less than the recommended fruit and 73.7% less than the recommended daily vegetable intake in 2009. This was lower than levels reported in 2000 for fruit intake, and represented no change for vegetable intake over this time period [34]. In comparison to these two countries with a similar prevalence of obesity, Australians in NSW are consuming more fruit and vegetables.

Population health surveys rely heavily on self-reported health information from respondents—with researchers trying to minimize respondent burden through the most parsimonious set of questions to meet the study objectives. Validity of short questions related to dietary practices, including fruit and vegetable intake, was demonstrated in the 1995 Australian National Nutrition Survey (ANNS), in which the questions were able to discriminate between groups with significantly different intakes of fruits or vegetables as compared to a 24 h recall in a sub-set of respondents, with more variability between sexes regarding understanding of a serving of vegetable [35]. Underestimation of food intake can be more prevalent in women, and may be extended to fruit and vegetable intake [36], so the gender differences found in our study may actually be larger than reported. Alternatively, our analyses cannot control for the effect of social approval bias that can affect the validity of both food frequency questionnaires and 24 h recall [37]. In addition to potential under and over-reporting, our results do not provide information about individual types and combination of fruits and vegetables consumed. This is a limitation in translation of these findings to the development of strategies to increase consumption of these food groups.

Regarding self-reported weights and height, men typically over-estimate height and women under-estimate weight in self-reported health measures [38,39,40]. The levels of overweight and obesity calculated in a recent study in Australia resulted in underestimates by up to five percent, as compared to measured values [41]. However, a validation study conducted on 45 and Up Study indicates excellent agreement between BMI categories based on self-reported and measured data (κ = 0.80) [42]. Other limitations include the cross-sectional design, which identifies only an association between sex, fruit and vegetable intake and body mass index. Furthermore, this study could not control for overall energy intake, which is an essential explanatory variable when looking at change in body weight. Further longitudinal data is required to better explain the risk of inadequate consumption, sex and overweight and obesity [43].

The large study sample should provide a robust approximation of how well cohort members, who constitute a substantial minority of the general population, are meeting these recommended daily nutritional intakes. The cross sectional nature of the study limits further interpretation related to the direction of the association. However, in future follow-ups of this cohort, direction of association between change in intake and body mass index can be further investigated.

## 5. Conclusions

These results suggest there is lack of a clear association between fruit and vegetable intake and weight status for both women and men—although the relationship appears stronger in women than men. Overweight and obese women, and obese men, were more likely than their normal weight counterparts to consume higher intakes of vegetables, as well as meet the recommended targets for combined fruit and vegetables, suggesting overconsumption of these foods as well as less nutrient-dense options result in energy imbalance. Overweight men, however, were less likely to consume high servings of both fruit and vegetables, and to meet dietary targets for fruit and vegetables compared to normal weight men, which suggests that public health approaches to increase fruit and vegetable intake may be beneficial in this group. Increased fruit consumption may be protective against weight gain in women, however the cross-sectional nature of the analysis limits further interpretation regarding positioning of fruit within overall dietary patterns of middle aged Australian women. Underweight men and women are at particular risk for low intakes of both fruit and vegetables. Analyses of additional health behaviors including physical activity, tobacco abuse and alcohol consumption (also available in the 45 and Up Study) may also illuminate important differences in health outcomes by sex and body weight. As obesity reflects energy imbalance, the rates of increase in weight among Australians needs to be addressed as this population continues to grow and age.
